# Root-knot nematodes produce functional mimics of tyrosine-sulfated plant peptides

**DOI:** 10.1073/pnas.2304612120

**Published:** 2023-07-10

**Authors:** Henok Zemene Yimer, Dee Dee Luu, Alison Coomer Blundell, Maria Florencia Ercoli, Paulo Vieira, Valerie M. Williamson, Pamela C. Ronald, Shahid Siddique

**Affiliations:** ^a^Department of Entomology and Nematology, University of California, Davis, CA 95616; ^b^Department of Plant Pathology and the Genome Center, University of California, Davis, CA 95616; ^c^U. S. Department of Agriculture-Agricultural Research Service Mycology and Nematology Genetic Diversity and Biology Laboratory, Beltsville, MD 20705

**Keywords:** PSY, root-knot nematode, root growth, plant-parasitic nematode, tyrosine-sulfated peptide

## Abstract

Root-knot nematodes (*Meloidogyne* spp.) are highly evolved obligate parasites threatening global food security. These parasites have a remarkable ability to establish elaborate feeding sites in roots, which are their only source of nutrients throughout their life cycle. A wide range of nematode effectors have been implicated in modulation of host pathways for defense suppression and/or feeding site development. Plants produce a diverse array of peptide hormones including *PLANT PEPTIDE CONTAINING SULFATED TYROSINE* (PSY)-family peptides, which promote root growth via cell expansion and proliferation. A sulfated PSY-like peptide RaxX (required for activation of XA21 mediated immunity X) produced by the biotrophic bacterial pathogen (*Xanthomonas oryzae* pv. *oryzae*) has been previously shown to contribute to bacterial virulence. Here, we report the identification of genes from root-knot nematodes predicted to encode PSY-like peptides (MigPSYs) with high sequence similarity to both bacterial RaxX and plant PSYs. Synthetic sulfated peptides corresponding to predicted MigPSYs stimulate root growth in Arabidopsis. *MigPSY* transcript levels are highest early in the infection cycle. Downregulation of *MigPSY* gene expression reduces root galling and egg production, suggesting that the MigPSYs serve as nematode virulence factors. Together, these results indicate that nematodes and bacteria exploit similar sulfated peptides to hijack plant developmental signaling pathways to facilitate parasitism.

## Significance

Plant peptides containing sulfated tyrosine (PSY)-family peptides are peptide hormones that promote root growth via cell expansion and proliferation. A PSY-like peptide produced by a bacterial pathogen has been shown to contribute to bacterial virulence. Here, we demonstrate that PSY-like peptides are encoded by a group of plant-parasitic nematodes known as root-knot nematodes. These nematode-encoded PSY mimics facilitate the establishment of parasitism in the host plant. Our findings are an example of a functional plant peptide mimic encoded by a phytopathogenic bacterium (prokaryote) and a plant-parasitic nematode (an animal).

Plant-parasitic nematodes (PPNs) are among the most destructive plant pathogens, causing an annual economic loss of $8 billion to US growers and over $100 billion worldwide (1, 2). Root-infecting sedentary endoparasitic nematodes including root-knot nematodes (RKNs; *Meloidogyne* spp.) and cyst nematodes (*Heterodera* spp. and *Globodera* spp.) cause the most significant economic damage. The RKN species *Meloidogyne incognita*, *Meloidogyne javanica*, and *Meloidogyne arenaria* have broad host ranges and can infect thousands of species, including annual and perennial crops and both dicots and monocots (3). Infective second-stage juveniles (J2s) of RKNs invade the plant close to the root tip. Once inside the root, J2s migrate between cells until they reach the vascular tissue (4), where they become immobile and induce the formation of several highly modified, adjacent multinucleated giant cells. While giant cells develop, neighboring cells hypertrophy and divide, leading to the formation of characteristic galls (root-knots) (5, 6). These feeding sites are the only source of nutrients for nematodes throughout their approximately 1-mo-long life cycle (7).

A wide range of nematode-secreted molecules have been implicated in establishing and maintaining feeding sites. Among these molecules are small peptides that resemble plant peptide hormones. The best studied of these “mimics” are CLE (CLAVATA3/embryo surrounding region)-like peptides produced by cyst nematodes. These CLE-like peptides are delivered as propeptides into the host cytoplasm through the nematode stylet. The propeptides are then post-translationally modified and proteolytically processed by host enzymes for secretion into the host’s extracellular space as mature CLE-like peptides. The N-terminal variable domain of the propeptide is required for this process (8–10). Nematode CLE-like peptides are thought to facilitate the formation of host feeding sites by activating CLE signaling pathways (11, 12). RKNs also encode mimics of CLE and other peptide hormones including CEP (C-terminally encoded peptides), RALF (rapid alkalization factors), and IDA (inflorescence deficient in abscission) peptides (11–17). In contrast to cyst nematode CLEs, the RKN peptide hormone mimics characterized so far lack an N-terminal variable domain and are thought to be directly delivered by the nematode to the host’s extracellular space following proteolytic cleavage of the secretion signal (11).

*PLANT PEPTIDE CONTAINING SULFATED TYROSINE* (PSY)-family peptides are secreted peptides that promote root growth via cell expansion and proliferation (18–20). PSY peptides have been identified in all higher plants and more recently in mosses and bacteria (19). PSY1 was identified from Arabidopsis suspension cell culture medium as an 18-amino acid tyrosine-sulfated glycopeptide derived from a 75-amino acid precursor peptide (18). Arabidopsis encodes eight homologs of PSY1 with conserved peptide domains (19, 20). Recently, three Arabidopsis leucine-rich repeat receptor kinases, plant peptide containing sulfated tyrosine receptor 1 (PSYR1), PSYR2, and PSYR3, that act as cognate receptors for all nine PSY homologs were identified (20). Experiments indicate that in Arabidopsis plants carrying a loss-of-function mutation in tyrosyl protein sulfotransferase (*tpst-1*), which likely leads to loss of function of all PSY family members, the PSYRs activate expression of genes encoding stress response transcription factors. Binding of PSY-family peptides to PSYRs represses constitutive signaling of this stress response, which allows for growth (20).

*Xanthomonas* species secrete a tyrosine-sulfated peptide RaxX (required for activation of XA21-mediated immunity X) with sequence similarity to a 13-amino acid motif conserved throughout plant PSYs (21). These RaxX peptides from *Xanthomonas* mimic the growth-promoting activities of PSYs (22). Competitive binding assays showed that both RaxX16 and RaxX21, synthetic derivatives of RaxX, compete effectively with AtPSY6 for binding to PSYR2 and PSYR3, indicating that RaxX is also perceived by Arabidopsis PSYRs (20).

The proposed role of PSY peptides in defense suppression, cell expansion and proliferation led us to hypothesize that PPNs might also produce and secrete PSY-like peptides into host plants. Here, we identify RKN genes encoding several PSY-like peptides with high sequence similarity to both bacterial RaxX and plant PSYs. We further characterize their expression and demonstrate their roles in plant growth and RKN parasitism.

## Results

### Root-Knot Nematode Genes Encode PSY-Like Peptides.

**Fig. 1.**
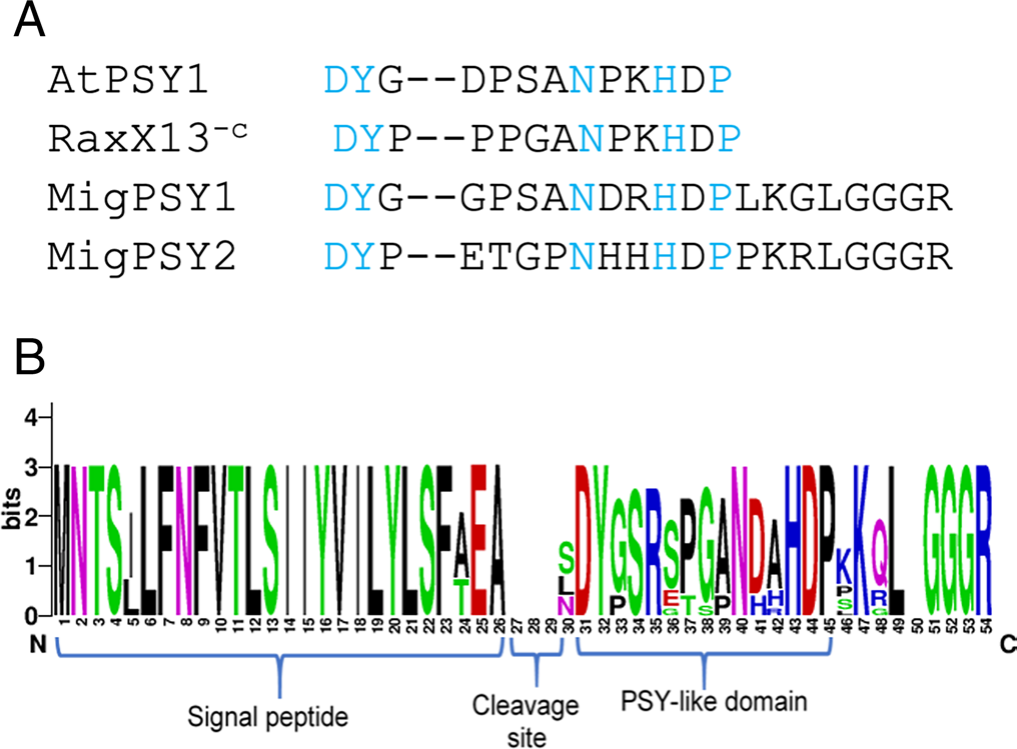
Root-knot nematodes are predicted to encode PSY-like peptides. (*A*) Amino acid sequences of the conserved PSY motif from Arabidopsis [*Arabidopsis thaliana*] AtPSY1 and from the bacterial species, *Xoo* (RaxX13^-c^), compared with the predicted translation products from the three MigPSY types. Letters in blue represent the conserved amino acids of the PSY motif. For each of the MigPSY types, the predicted product extending from the conserved motif to the C terminus is shown. (*B*) Protein logo alignment of 11 predicted *MigPSY* gene products in the species *M. incognita*, *M. javanica*, and *M. arenaria* (*SI Appendix*, Table S1). Spaces with no amino acids indicate positions that are not present in the majority of the predicted products. The amino acid sequences are predicted to contain a highly conserved N-terminal secretion signal peptide followed by a PSY-like domain beginning 1 to 4 amino acids after the signal peptide cleavage site.

We used the conserved 13-amino acid PSY domain of RaxX (RaxX13^-C^) to interrogate predicted translation products of cDNA sequences from available nematode databases (23, 24). This conserved motif contains a tyrosine residue immediately preceded by an aspartic acid and followed by the amino acid sequence NXXHXP downstream (Fig. 1*A*). The aspartic acid–tyrosine residue pair is the minimum requirement for tyrosine sulfation (25), a post-translational modification critical for PSY activity (18). Following reinterrogation with initial hits, we identified 11 cDNA translation products (*SI Appendix*, Table S1) in the closely related RKN species *M. incognita*, *M. javanica*, and *M. arenaria*. As *M. incognita*, *M. javanica*, and *M. arenaria* are closely related asexual species that belong to the *M. incognita* group, we will refer to the nematode genes as *MigPSY*s (*M. incognita* group PSYs) thereafter.

Based on differences in amino acid residues within the conserved domain, we classified MigPSYs into three types: MigPSY1, MigPSY2, and MigPSY3 (Fig. 1*A*). MigPSY1 was identified in only one isolate of *M. arenaria,* while MigPSY2 was present in *M. arenaria* and *M. javanica* but not in *M. incognita*. For MigPSY3, 2 amino acids (SR) were inserted between the conserved DY and NXXHXP motifs. MigPSY3 was the most widely represented, with two to three copies present in the genome of each MIG species. We also identified closely related PSY-like motifs in genome drafts of additional Clade I *Meloidogyne* species (*M. enterolobii*, *M. floridensis*, and *M. luci*) but not in more distantly related *Meloidogyn*e spp. or in other groups of PPNs.

All identified *MigPSYs* are predicted to produce primary translation products of 50 to 51 amino acids (Fig. 1*B* and *SI Appendix*, Table S1). Each is predicted to be cleaved after a highly conserved N-terminal signal peptide, resulting in a 21 to 24 amino acid peptide with the conserved PSY motif at or very close to its N terminus. For MigPSY1 and MigPSY2, there is a 4 amino acid YTIN domain between the predicted signal peptide cleavage site and the conserved domain, whereas MigPSY3s have only a single aa (S/L). No transmembrane domain was predicted by DeepTMHMM analysis (26). Without additional processing, each translation product would contain a C-terminal GGGR sequence (Fig. 1*B* and *SI Appendix*, Table S1). Comparison to genomic sequence revealed that, for all 11 genes, there is one intron, and the two exons are spliced together upon transcript processing to constitute the aspartate residue that initiates the PSY-like domain. MigPSYs, like other RKN peptide mimics (IDA, CEP, RALF, and CLE), lack a variable domain and may therefore be directly delivered to the host’s extracellular space (12).

### Exogenous Application of MigPSY Peptides Enhances Plant Root Growth.

**Fig. 2.**
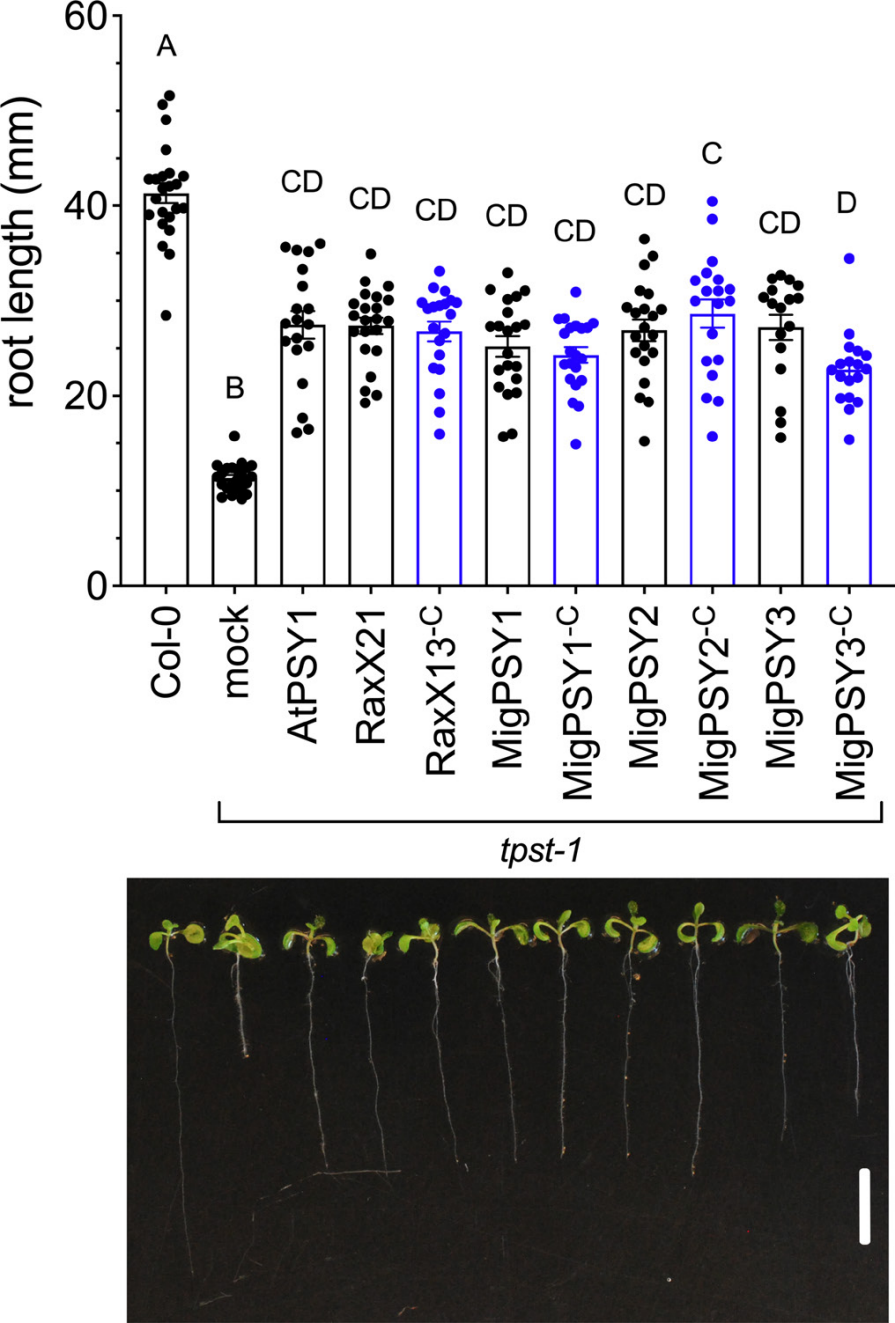
Exogenous treatment with MigPSY peptides promotes root growth. Arabidopsis Col-0 or *tpst-1* mutant seeds were sown on MS media containing either water (mock) or 100 nM of synthetic tyrosine-sulfated peptide derived from the predicted MigPSY mature sequence (black) or just the conserved DYX_5-7_NXXHXP PSY-like sequence (denoted by ^-C^, blue). AtPSY1 from Arabidopsis and RaxX21 and RaxX13 from *Xoo* were used as positive controls. Bars represent the mean root length (mm) 9 d after sowing ±SEM of at least 17 seedlings, shown as individual dots (*Top*). Statistical significance was analyzed using Tukey’s multiple comparisons test with different letters representing significant differences (*P* ≤ 0.05). Representative seedlings are shown in the bottom panel (Scale bar: 1 cm). Data represent one of three independent experiments with similar results.

Based on our bioinformatic analysis, we hypothesized that the nematode peptides would be secreted and that these peptides would have PSY peptide-like activity. To test this hypothesis, we measured the growth response of Arabidopsis roots after peptide treatment. For these experiments, we utilized *tpst-1* mutant plants, which have dysfunctional tyrosine sulfation, a crucial post-translational modification for PSY activity (27). The *tpst-1* mutant displays a stunted root phenotype that can be partially rescued by exogenous treatment with sulfated PSY or RaxX peptides (Fig. 2) (21). In the current study, we assessed the root growth response of *tpst-1* mutant seedlings to treatment with synthetic peptides corresponding to the predicted mature peptide sequence of each of the three MigPSY types. Responses to the previously characterized peptides AtPSY1, RaxX21 (a 21-amino acid derivative of RaxX from *Xanthomonas oryzae* pv. *oryzae*), and RaxX13^-c^, which contains only the conserved 13-amino acid PSY-like domain, were included as controls (21). All peptides were sulfated at their N-terminal tyrosine (*SI Appendix*, Table S2). We grew seedlings on Murashige and Skoog (MS) medium containing individual synthetic peptides provided at a concentration of 100 nM and measured root lengths 9 d after sowing. Each of the MigPSY peptides significantly increased the root length of *tpst-1* Arabidopsis seedlings to an extent similar to plants treated with AtPSY1, RaxX21, and RaxX^-c^ (Fig. 2). In addition, truncated MigPSY peptides containing only the conserved DYX_5-7_NXXHXP PSY-like domain (MigPSY1^-C^, MigPSY2^-C^, and MigPSY3^-C^) also promoted *tpst-1* root growth. However, while roots treated with the MigPSY1^-C^ and MigPSY2^-C^ peptides obtained lengths similar to those treated with the longer peptides, treatment with the truncated MigPSY3^-C^ peptides caused a smaller increase compared to treatment with the longer MigPSY3 peptide.

### *MigPSY* Gene Expression Is Highest at Early Stages of Nematode Infection.

**Fig. 3.**
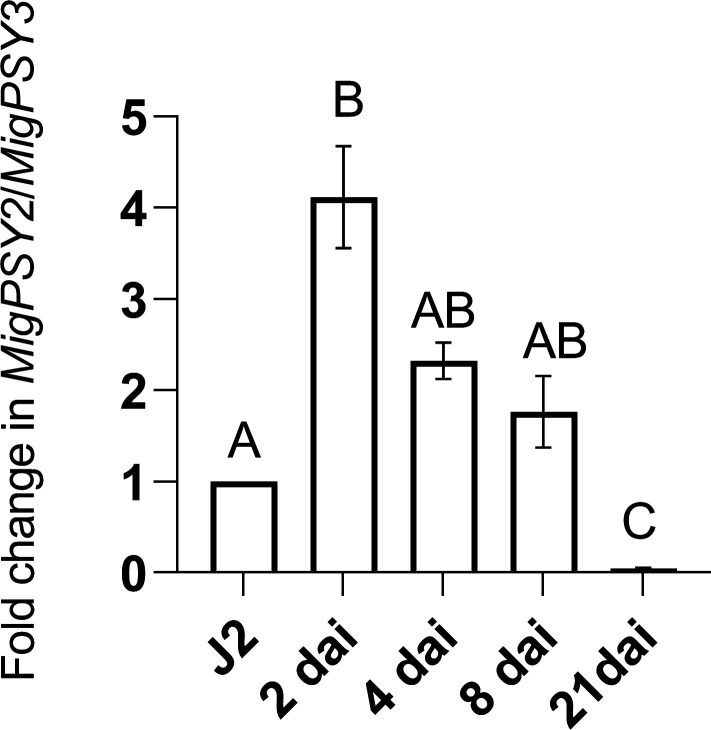
*MigPSY* expression is induced during early stages of nematode infection. Relative expression levels of *MigPSY* genes in *M. javanica* following inoculation of tomato roots at the indicated time points after inoculation, as determined by qRT-PCR. Values represent relative expression levels with the level in preparasitic second-stage juveniles (J2s) set to 1. The mRNA levels were measured as three technical replicates per sample. The transcript levels of each gene were normalized to that of the nematode housekeeping gene *β-actin-1* and *EF-1α* with three biological replicates each (*n* = 3). Data are presented as the mean ± SE. Each biological replicate contained a pool of hundreds of small root segments with infection sites for each post inoculation time point. dai, days after inoculation. Statistical significance was analyzed using test Holm-Sidak’s multiple comparison test. Different letters represent significant differences (*P* ≤ 0.05).

We analyzed the relative expression levels of *MigPSY* in preparasitic juveniles and during infection. For this experiment, we used *M. javanica* (28). *M. javanica* can infect over a thousand host species, including tomato (*Solanum lycopersicum*) and rice (*Oryza sativa*). Database searches indicated that the *M. javanica* genome harbors one copy of *MigPSY2* and two closely related copies of *MigPSY3* but does not encode *MigPSY1* (*SI Appendix*, Table S1). To investigate the expression pattern of *MigPSYs* during host infection, we designed qPCR primers that can efficiently amplify both *MigPSY2* and *MigPSY3* (*SI Appendix*, Fig. S1). We collected several hundred root segments containing galls from tomato and rice plants inoculated with *M. javanica* at four time points after inoculation (2, 4, 8, and 21 days after inoculation [dai]). We selected these time points to represent the following stages of parasitism: entry into the root and migration to the feeding site (2 dai); the initial formation of giant cells and galls (4 dai); third molt (J3) nematodes (8 dai); and final molt into adult females (21 dai). Compared to their expression levels in preparasitic juveniles, *MigPSY* expression levels were higher at 2 dai (Fig. 3 and *SI Appendix*, Fig. S2). However, *MigPSY* expression decreased at 4 and 8 dai and was barely detectable at 21 dai. These results are consistent with *MigPSY* expression patterns in *M. incognita* across developmental stages on tomato as reported by Blanc-Mathieu et al. (23). The induced expression of *MigPSYs* suggests that their gene products may be involved in facilitating the early stages of nematode infection.

### In Situ Hybridization Localizes *MigPSY* Transcripts to the Esophageal Gland Region of Preparasitic Juveniles.

**Fig. 4.**
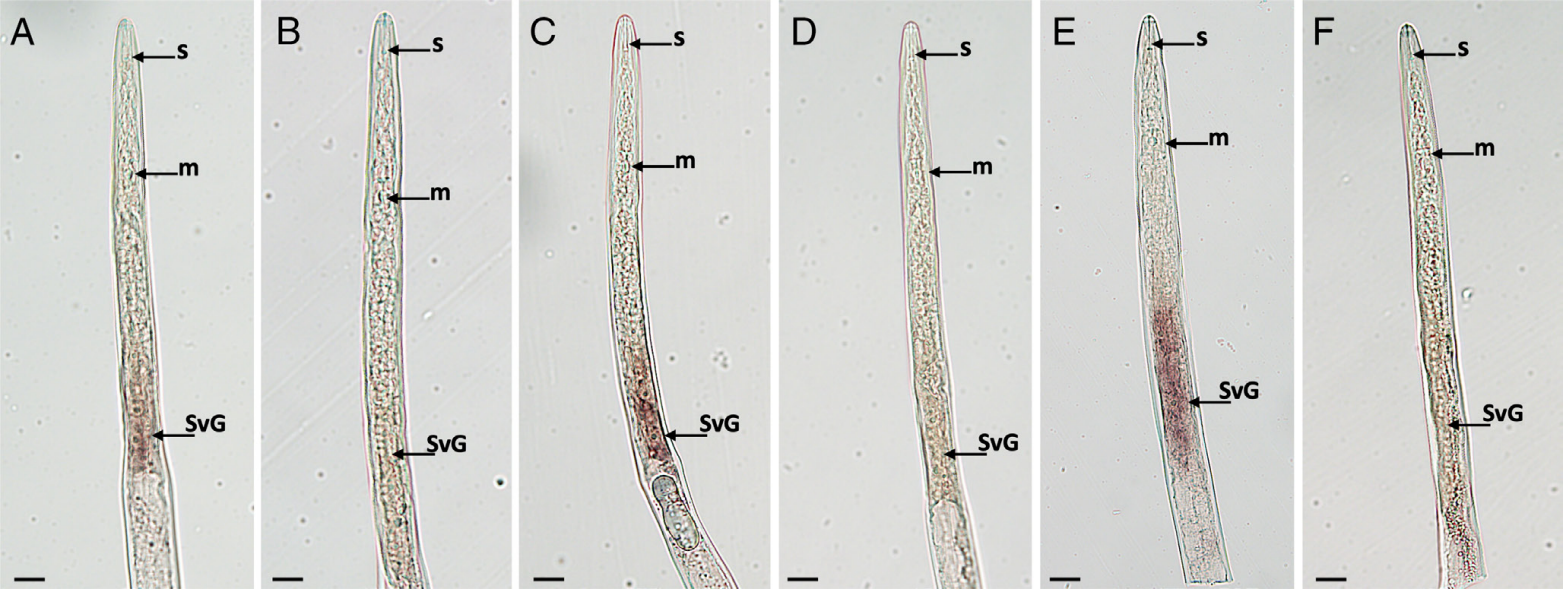
In situ hybridization of *MigPSY* transcripts in *M. javanica* and *M. incognita* infective juveniles. (*A*) Positive detection of *MigPSY2* transcripts by an antisense DIG-labeled DNA probe in the subventral glands of *M. javanica.* (*B*) A sense DIG-labeled DNA probe, used as a negative control for *MigPSY2* in *M. javanica*, showed no detectable labeling. (*C*) Positive detection of *MigPSY3* transcripts by an antisense DIG-labeled DNA probe in the subventral glands of *M. javanica.* (*D*) A sense DIG-labeled DNA probe, used as a negative control for *MigPSY3* in *M. javanica*, showed no detectable labeling. (*E*) Positive detection of *MigPSY3* transcripts by an antisense DIG-labeled DNA probe in the subventral glands of *M. incognita*. (*F*) A sense DIG-labeled DNA probe, used as a negative control for *MigPSY3* in *M. incognita*, showed no detectable labeling. Abbreviations: DIG, digoxigenin; s, stylet; m, metacorpus; SvG, subventral glands. Procedure was repeated three times independently. Representative images are shown. (Scale bars, 10 µm).

We used in situ hybridization to localize *MigPSY2* transcripts in preparasitic J2s of *M. javanica* (Fig. 4 *A* and *B*) *and MigPSY3* transcripts in preparasitic J2s of *M. javanica* (Fig. 4 *C* and *D*) and *M. incognita* (Fig. 4 *E* and *F*). We detected a specific signal limited to the subventral esophageal glands when hybridized with a DIG-labeled antisense probe for both *MigPSY2* and *MigPSY3* (Fig. 4 *A*, *C*, and *E*). We observed no hybridization when using a DIG-labeled sense probe as negative control for both species (Fig. 4 *B*, *D*, and *F*). The subventral esophageal gland cells are where secretions that are delivered into the host during early stages of parasitism are synthesized and have been shown to be most active at this stage (29). Based on these observations, we hypothesize that MigPSYs are produced in nematode subventral glands from where they are released into the host.

### Reducing Expression of *MigPSYs* in Preparasitic J2s Leads to Fewer Galls on Rice Plants.

**Fig. 5.**
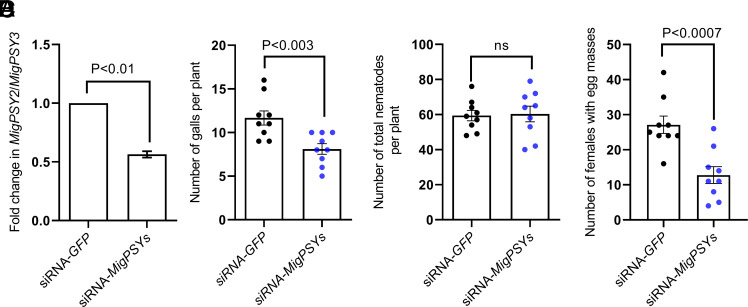
MigPSYs have a role in root-knot nematode parasitism of rice. (*A*) Relative transcript abundance of *MigPSYs* in preparasitic J2s soaked in siRNA targeting either *MigPSYs* or *GFP*. Bars represent mean ± SEM from three independent experiments (*n* = 3). Significant differences were assessed using Student’s *t* tests (two-sided; *P* < 0.05). (*B*) Number of galls present per rice root system infected with J2s soaked in siRNA targeting either *MigPSYs* or *GFP* at 30 dai. (*C*) Average number of females with egg masses per rice root system infected with J2s soaked in siRNA targeting either *MigPSYs* or *GFP* at 30 dai. (*D*) Total number of nematodes, including all developmental stages, per rice root system infected with J2s soaked in siRNA targeting either *MigPSYs* or *GFP* at 30 dai. In (*B–D*), approximately 1,000 *M. javanica* infective juveniles were inoculated onto 14-d-old rice seedlings. Experiments were performed three times independently with similar outcomes. Data from one experiment are shown. Significant differences were assessed using Student’s *t* tests (two-sided; *P* < 0.05). Source data from all three experiments are included as *SI Appendix*. MigPSY, *M. incognita* group plant peptide containing sulfated tyrosine; siRNA, small-interfering RNA; J2, preparasitic second-stage juvenile; GFP, green fluorescent protein; dai, days after inoculation; ns, not significant (*P* > 0.05).

To investigate the possible roles of MigPSY in parasitism, we soaked preparasitic J2 of *M. javanica* in a short interfering RNA (siRNA) solution targeting all *MigPSY*s present in *M. javanica* (*SI Appendix*, Fig. S1). A siRNA targeting the green fluorescent protein (*GFP*) gene was used as a control treatment. qRT-PCR showed that relative *MigPSY* transcript levels were lower in J2s treated with the *MigPSY* siRNA compared to control J2s (Fig. 5*A*). We inoculated rice plants with siRNA-treated nematodes and examined their roots at 30 dai. The average number of galls on plants inoculated with J2s soaked in siRNA targeting *MigPSYs* was significantly lower than those inoculated with control J2s (Fig. 5*B*) and had significantly fewer females with egg masses (Fig. 5*C*). However, the total number of nematodes within the roots, which includes nematodes of different developmental stages, did not differ between roots inoculated with J2s soaked in siRNA targeting *MigPSYs* and control roots (Fig. 5*D*). Taken together, these results indicate that the reduced *MigPSY* transcript levels did not affect the ability of J2 to locate and infect rice roots but did compromise gall formation and development of nematodes into mature egg-laying females.

## Discussion

In the present study, we report the identification and characterization of genes encoding PSY-family peptides from RKN. Several lines of evidence support a role for MigPSYs in parasitism by *M. javanica*. Transcripts are highest in the early stages of parasitism and are localized to subventral glands, the major route and origin for secretion of effectors through the stylet in the early stages of infection. Silencing PSYs compromises parasitism; we show that fewer galls are produced, and nematode development is compromised in rice following RNAi treatments that reduce MigPSY transcript levels. PSY mimics have been linked to *Xanthomonas* spp. pathogenicity. Even though RKN and *Xanthamonas* spp. have completely different lifestyles, both are biotrophic pathogens that cause hypertrophy of host tissue. Hence, RKN and *Xoo* may use PSY mimics to change host environments to facilitate parasitism. These pathogen types presumably acquired/evolved PSY peptides independently, with their sequences, activity, and expression patterns tailored for each pathogen.

It is intriguing that closely related PSY-like motifs are identified only in the MIG species and a few other species in Clade 1 of *Meloidogyne* and not in more distantly related *Meloidogyne* spp. or other groups of plant parasitic nematodes. This suggests that functional PSY mimics have only been recently acquired in nematodes. MIG species have broad host ranges and are globally the most damaging group of plant parasitic species to agricultural crops (3). It is tempting to speculate that these PSY mimics may have had a role in this success.

So far, we know little about the post-translational processing of MigPSYs. This is likely to be a challenge as the host interaction zone for these obligate parasites is very small and difficult to access. For all MigPSY genes, the transcript is predicted to be translated into a short peptide product with a very conserved secretion signal that is post-translationally cleaved 1 to 4 amino acids before the DY residues. While tyrosine sulfation is not generally present in bacteria aside from *Xanthomonas*, tyrosyl protein sulfotransferase (TPST) is ubiquitous in eukaryotic species, both plant and animal. Two genes encoding TPST are present in *C. elegans* and a conserved homolog is present in the genomes of Mig species (e.g., Minc3s00073g03623). Thus, it seems likely that PSY peptides are sulfated by the nematode prior to secretion.

Intriguingly, the conserved domain of MigPSY3 has a two-amino-acid insertion that to the best of our knowledge is not present in any known plant PSY. The truncated synthetic peptide MigPSY3^-C^ stimulated root elongation in our Arabidopsis assay to a lesser extent than the similarly truncated peptides for MigPSY1 or MigPSY2 suggesting that the two aa insertion may alter binding to the plant receptor. However, MigPSY3 is the most widespread and conserved among the closely related MIG species, suggesting that it has a valuable role in this highly successful group of parasites. Additional work will be required to understand the contribution of MigPSYs in defense suppression, gall formation, and nematode reproduction. Similarly, the presence of other post translational modifications such as hydroxylation and the role of the conserved amino acid residues at the C terminus of the MigPSY peptides that are specific to RKN-encoded PSYs remain to be investigated.

The recent discovery that receptors of endogenous Arabidopsis PSY peptides constitutively activate expression of stress response genes and that this expression is reduced in plants treated with the PSY5 ligand suggests a possible role for bacterial and nematode PSYs in plant pathogenicity. These ligands may bind to and repress host defenses activated by PSYRs, thus facilitating parasitism and feeding site development. However, this may not be the whole story, and additional studies on the interaction of pathogen PSYs with host receptors is needed. Further studies are needed to assess if the recently identified Arabidopsis PSYR receptors are involved in perception of MigPSYs. Furthermore, it is unclear if MigPSY is recognized by the rice XA21 immune receptor, which specifically detects and generates an immune response to RaxX but not to plant PSY.

## Materials and Methods

### Gene Identification and Characterization.

We used the following databases to identify nematode genes and transcripts described in this work: https://www6.inrae.fr/meloidogyne_incognita; https://parasite.wormbase.org/index.html. Translation products were characterized for signal peptide sequences using SignalP-6.0 (https://services.healthtech.dtu.dk/service.php?SignalP), and the presence of any transmembrane domain was assessed by DeepTMHMM analysis https://doi.org/10.1101/2022.04.08.487609).

Sequence logos were generated using WebLogo (http://weblogo.berkeley.edu/).

### Plant Growth and Nematode Inoculation.

Pure cultures of *M. javanica* strain VW4 were maintained on *Solanum lycopersicum* cv. Momar Verte plants grown in sand in a greenhouse (30). Nematode eggs were collected from 3-mo-old cultures, and preparasitic J2s were hatched as described (31) with minor modifications. Seeds of rice (*Oryza sativa* L.) subsp. *japonica* “Kitaake” and tomato (*Solanum lycopersicum*) cultivar “Moneymaker” were surface sterilized in 70% (v/v) ethanol and 4% sodium hypochlorite for 30 min and germinated on wet filter paper at 28 °C in darkness for 3 d. Seedlings were then transplanted and grown in Sand Absorbent Polymer (SAP) substrate in a growth chamber (26 °C; 12 h light/12 h dark regime). Plants were fertilized with Hoagland solution three times a week (32). Two-week-old rice and tomato seedlings were inoculated with 2,000 J2s of *M. javanica* or mock-inoculated with water as a control. Nematode infection was evaluated 30 dai as described (33).

### RNA Extraction, cDNA Synthesis, and qRT-PCR.

For gene expression analysis of *MigPSYs* during *M. javanica* parasitism of tomato and rice, plants were grown in sand and inoculated with nematodes as above. The entire root (for rice) and root tips or nematode galls (for tomato) were harvested at 2, 4, 8, and 21 dai with *M. javanica.* Samples were immediately frozen in liquid nitrogen and stored at –80 °C. Total RNA was extracted using an RNeasy Plant Mini Kit following the manufacturer’s instructions. RNA concentration and purity were measured using a NanoDrop OneC Microvolume UV-Vis Spectrophotometer (Thermo Scientific). TURBO DNase treatment was carried out to remove genomic DNA from total RNA samples (TURBO DNA-free Kit™). One-step qRT-PCR was performed using an iTaq Universal SYBR Green One-Step qRT-PCR Kit (BIO-RAD). The reaction was performed in a total volume of 20 µL by mixing 10 µL iTaq universal SYBR Green reaction, 0.25 µL of iScript reverse transcriptase, 1 µL of each primer (10 µM), 5.75 µL nuclease-free water, and 1.5 µL (80 ng µL^–1^) DNase-treated RNA. The reaction was carried out with an Applied Biosystem QuantStudio 3 Real-Time PCR System using the following conditions: reverse transcription at 50 °C for 10 min, polymerase activation at 95 °C for 1 min, followed by 40 cycles of denaturation at 95 °C for 15 s, and annealing/extension and plate reading at 60 °C for 60 s. A melting curve analysis was conducted by gradually increasing the temperature to 95 °C. The expression analysis was performed in triplicate using three independent biological samples, consisting of a pool of eight plants each. The transcript levels of *MigPSY*s were normalized to those of the nematode housekeeping gene *β-actin-1* and *EF-1α* in the tomato experiment and *β-actin-1* in the rice experiment (34, 35). Relative gene expression data were computed according to Pfaffl (36).

### Root Growth Assays.

*Arabidopsis thaliana* Col-0 and *tpst-1* mutant seeds (SALK_009847) were surface-sterilized for 10 min with 70% (v/v) ethanol and then stratified in 0.1% agarose in the dark (4 °C) for 3 to 4 d. Solid nutrient media plates were prepared with 1X Murashige and Skoog (MS) medium with vitamins (MSP09; Caisson Labs), 1% sucrose, pH 5.7, and 0.3% Gelzan (G024; Caisson Labs). Tyrosine-sulfated peptides (listed in *SI Appendix*, Table S2) were synthesized by the Protein Chemistry Facility at the Gregor Mendel Institute and resuspended in double-distilled water. Synthetic peptide (or water for mock treatments) was added to the medium to a final concentration of 100 nM just before pouring into a 100 mm × 15 mm Petri dish. Seeds were distributed equally on the surface of the medium along a row 2 to 3 cm from the upper edge of the Petri dish and the lids were secured with Micropore surgical tape. Plates were incubated vertically in a 21 °C chamber with 16-h-light/8-h-dark photoperiod. Seedlings with delayed germination were marked after 3 d and were not included in the analysis. Root lengths (mm) were measured 9 d after sowing using ImageJ software (version 1.53, NIH).

### In Situ Hybridization.

Total RNA was extracted from preparasitic J2s of both *M. javanica* and *M. incognita* using RNeasy Plant Mini Kit (Qiagen) and the first-strand cDNA synthesized with High-Capacity cDNA Reverse Transcription Kits (Applied Biosystems, Thermo Scientific). The cDNA template was then used to PCR amplify 79 bp in the CDS region of *MigPSY2* (*M. javanica*) and 81 and 88 bp in the CDS region of *MigPSY3 (M. javanica* and *M. incognita).* The primers used for probe synthesis are listed in *SI Appendix*, Table S3. The in situ hybridization protocol was adopted from Gao et al. (37). A sense (negative control) and antisense single-strand cDNA probe were synthesized in two independent asymmetric PCR reactions with digoxigenin (DIG) labeling mix (1mM dATP, 1mM dCTP, 1mM dGTP, 0.65mM dTTP, 0.35mM DIG-dUTP; Roche, CA, USA). Preparasitic J2s of *M. javanica* and *M. incognita* were fixed in 2% paraformaldehyde in M9 buffer for 18 h at 4 °C, followed by 4 h of incubation at room temperature. The fixed preparasitic J2s were mechanically cut into fragments and enzymatically permeabilized with proteinase K (0.5 mg/mL) solution (Roche). Hybridization with sense (negative control) or antisense dig-labeled probes was performed in separate nematode samples overnight at 50 °C. Hybridized probes were detected by anti-DIG antibodies conjugated with alkaline phosphatase enzyme (anti-DIG-AP) (Roche). Photographs were captured using an Olympus BX51 compound microscope using a DP74 Olympus camera.

### Gene Silencing.

*MigPSY* transcript levels were knocked down using an siRNA as described previously (38). Briefly, a 21-nt long target sequence in the *MigPSY3* mRNA beginning with an AA dinucleotide was identified. Sense and antisense oligonucleotides for the 21-nt target sequence were obtained in which the U’s were substituted with T’s. The 8-nt long sequence (5′-CCTGTCTC-3′) complementary to the T7 promoter primer was added to the 3′ ends of both the sense and antisense oligonucleotides. Afterward, a double-stranded RNA (dsRNA) was synthesized from the sense and antisense oligonucleotides using a Silencer® siRNA Construction Kit (Cat. Nr. AM1620) according to the manufacturer’s instructions. The siRNA targeting *eGFP* was used as a control. siRNA yields were measured using a NanoDrop OneC Microvolume UV-Vis Spectrophotometer (Thermo Scientific). Approximately, 10,000 freshly hatched preparasitic J2s were incubated in 50 μL solution containing 200 ng/µL of siRNA, 3 mM spermidine (Sigma-Aldrich), and 50 mM octopamine-HCl (Sigma-Aldrich). After a 24-h incubation at 26 °C in the dark, nematodes were washed with sterile water several times. J2s were then incubated for another 24 h in sterile water before sampling for RNA extraction. Total RNA was extracted from J2s subjected to siRNA targeting either *MigPSY* or *GFP*, and one-step qRT-PCR was performed as described above to analyze the *MigPSY* transcript levels. For infection assay, rice seeds were germinated and grown in sand as described above. Two-week-old rice seedlings were inoculated with 1,000 J2s. Two days post-inoculation, plants were transferred to a hydroponic system to synchronize the infection. In brief, plants were carefully uprooted from the sand substrate, washed under tap water to remove the adhering sand particles, and transferred to 1/4 strength Hoagland’s solution. Plants were maintained under hydroponic conditions for 28 d, and nematode infection was evaluated 30 dai with *M. javanica*. Roots were stained with acid fuchsin to stain nematodes. Nematodes inside roots were enumerated by microscopic examination. Statistical analysis and graphical representations were performed using GraphPad Prism software version 8.3.0.

## Supplementary Material

Appendix 01 (PDF)

Dataset S01 (XLSX)

## Data Availability

All study data are included in the article and/or supporting information.
