# Supplementary material for: Root-knot nematodes produce functional mimics of tyrosine-sulfated plant peptides
Source: Proc Natl Acad Sci U S A. 2023 Jul 10;120(29):e2304612120. doi: 10.1073/pnas.2304612120 (PMC10629525; doi:10.1073/pnas.2304612120)
Supplement: Supplementary file 1 — Appendix 01 (PDF) [file pnas.2304612120.sapp.pdf]

PNAS

Supporting Information for
Root-knot nematodes produce functional mimics of tyrosine-sulfated plant peptides

Henok Zemene Yimer, Dee Dee Luu, Alison Coomer Blundell, Maria Florencia Ercoli, Paulo Vieira, Valerie M. Williamson, Pamela C. Ronald, and Shahid Siddique

Corresponding authors: Shahid Siddique and Pamela C. Ronald
Email: ssiddique@ucdavis.edu and pcronald@ucdavis.edu

This PDF file includes:

Figure S1 to S2
Tables S1 to S3
Legend for Dataset S1

Other supporting materials for this manuscript include the following:

Dataset S1

```
PSY2     ATGAATACATCTCTCTTATTTAATTTTGTTACACTCTCAATTATTTATGTTATTCTTTAC 60
PSY3b    ATGAATACATCTATCTTATTTAATTTTGTTACACTCTCAATTATCTATGTTATTCTTTAC 60
PSY3a    ATGAATACATCTATCTTATTTAATTTTGTTACACTCTCGATTATTTATGTTATTCTTTAC 60

                                siRNA                       qPCR-F

PSY2     TTATCATTCGCTGAAGCATACACCATAAATGATTATCCTGAGACTGGTCCAAATCATCAT 120
PSY3b    TTATCCTTCACGGAAGCATCGGATTATGG---AAGCCGTAGCCCTGGTGCAAATGATGCA 117
PSY3a    TTATCATTCGCTGAAGCATTAGATTATGG---AAGCCGTAGCCCTGGTGCAAATGATGCA 117


PSY2     CATGATCCACCAAAACGATTAGGAGGAGGACGATAA 156
PSY3b    CATGATCCAAAAAAACAATTAGGAGGAGGACGTTAG 153
PSY3a    CATGATCCATCAAAACAATTAGGAGGAGGACGTTAG 153

                                qPCR-R
```

Fig. S1. CDS alignment of MigPSYs from *Meloidogyne javanica.* Highlighted regions are siRNA target site (red) and qPCR primer sites (green).

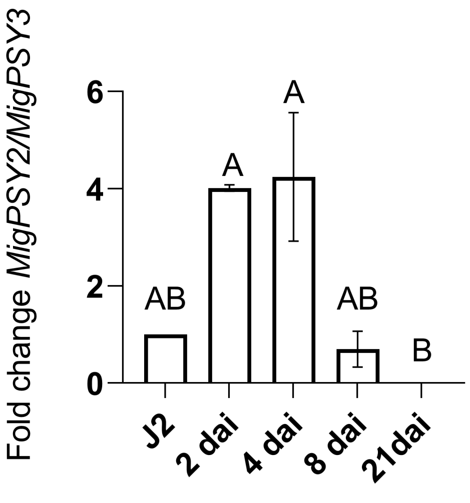

Fig. S2. *MigPSY* expression during nematode infection on rice. Relative expression levels of *MigPSY* genes in *M. javanica* following inoculation of rice roots at the indicated time points after inoculation, as determined by reverse transcription quantitative PCR (RT-qPCR). Values represent relative expression levels with the level in pre-parasitic second-stage juveniles (J2s) set to 1. The mRNA levels were measured as three technical replicates per sample. The transcript levels of each gene were normalized to that of the nematode housekeeping gene *β-actin-1* with two biological replicates each (n=2). Data are presented as the mean ± SE. Each biological replicate contained a pool of hundreds of small root segments with infection sites for each post inoculation time point. dai, days after inoculation. Statistical significance was analyzed using test Holm-Sidak's multiple comparison test. Different letters represent significant differences ($P \leq 0.05$).

Table S1. PSY-like peptides encoded in MIG species. Peptides followed by the same letter are identical in sequence. Underlined letters represent a highly conserved N-terminal signal peptide. Letters in red represent a conserved tyrosine residue and letters in blue represent a PSY-like domain with additional C-terminal GGGR sequence.

cDNA designation	MigPSY type	# Amino acids in encoded peptide	Predicted peptide length after signal cleavage	Peptide sequence
M.Arenaria_Scaff18626g092470	MaPSY1	51	21	MNTSLLFNFVTLSIIYVILYLSFAEAYTINDYG--GPSANDRHDPLKGL-GGGR
M.Arenaria_Scaff5652g054995	MaPSY2	51	21	MNTSLLFNFVTLSIIYVILYLSFAEAYTINDYP--ETGPNHHHDPPKRL-GGGR
M.Javanica_Scaff4941g037068	MjPSY2	51	21	MNTSLLFNFVTLSIIYVILYLSFAEAYTINDYP--ETGPNHHHDPPKRL-GGGR
M.Arenaria_Scaff13461g082170	MaPSY3[a]	50	23	MNTSILFNFVTLSIIYVILYLSFTEA---SDYGSRSPGANDAHDPKKQL-GGGR
M.Arenaria_Scaff5456g053959	MaPSY3[b]	50	23	MNTSILFNFVTLSIIYVILYLSFAEA---LDYGSRSPGANDAHDPKKQL-GGGR
M.Arenaria_Scaff20106g094880	MaPSY3[c]	50	23	MNTSILFNFVTLSIIYVILYLSFAEA---LDYGSRSPGANDAHDPSKQL-GGGR
Minc3s01748g26028	MiPSY3[a]	50	23	MNTSLLFNFVTLSIIYVILYLSFAEA---SDYGSRSPGANDAHDPKKQLRGGGR
Minc3s07420g41102	MiPSY3[b]	50	23	MNTSILFNFVTLSIIYVILYLSFTEA---SDYGSRSPGANDAHDPKKQL-GGGR
Minc3s02310g29383	MiPSY3[c]	51	24	MNTSILFNFVTLSIIYVILYLSFTEA---SDYGSRSPGANDAHDPKKQL-GGGR

M.Javanica_Scaff5264g038601	MjPSY3[a]	50	23	MNTSILFNFVTLSIIYVILYLSFAEA---LDYGSRSPGANDAHDPSKQL-GGGR
M.Javanica_Scaff14581g070241	MjPSY3[b]	50	23	MNTSILFNFVTLSIIYVILYLSFTEA---SDYGSRSPGANDAHDPKKQL-GGGR

Table S2. Synthetic peptide sequences.

Peptide	Sequence
AtPSY1	DY(SO_3)GDPSANPKHDPGVPPS
RaxX21	HVGGGDY(SO_3)PPPGANPKHDPPPR
RaxX13^{-C}	DY(SO_3)PPPGANPKHDP
MigPSY1	DY(SO_3)GGPSANDRHDPLKGLGGGR
MigPSY1^{-C}	DY(SO_3)GGPSANDRHDP
MigPSY2	DY(SO_3)PETGPNHHHDPPKRLGGGR
MigPSY2^{-C}	DY(SO_3)PETGPNHHHDP
MigPSY3	DY(SO_3)GSRSPGANDAHDPKKQLGGGR
MigPSY3^{-C}	DY(SO_3)GSRSPGANDAHDP

Table S3. Primer sequences used in this study.

Primer name	Sequence
MjPSY3_ISH_F	CGCTGAAGCATTAGATTATGGA
MjPSY3_ISH_R	CGTCCTCCTCCTAATTGTTTTG
MiPSY3_ISH_F	CATTCGCTGAAGCATCAGATT
MiPSY3_ISH_F	CGTCCTCCTCCTCTTAATTGTT
MjPSY2_ISH_F2	CATTCGCTGAAGCATACACC
MjPSY2_ISH_R	CCTAATCGTTTTGGTGGATCA

Dataset S1 (separate file). Raw data for Figures 2 and 5.
